# Trends in external causes of child and adolescent mortality in Poland, 1999–2012

**DOI:** 10.1007/s00038-016-0908-7

**Published:** 2016-10-20

**Authors:** Aneta Grajda, Zbigniew Kułaga, Beata Gurzkowska, Magdalena Góźdź, Małgorzata Wojtyło, Mieczysław Litwin

**Affiliations:** 1 Department of Public Health, The Children’s Memorial Health Institute, Warsaw, Poland; 2 Department of Nephrology and Arterial Hypertension, The Children’s Memorial Health Institute, Warsaw, Poland

**Keywords:** External causes of death, Children, Adolescents, Annual percent change

## Abstract

**Objectives:**

To examine the pattern and trend of deaths due to external causes among Polish children and adolescents in 1999–2012, and to compare trends in Poland’s neighboring countries.

**Methods:**

Death records were obtained from the Central Statistical Office of Poland. External causes mortality rates (MR) with 95 % confidence interval were calculated. The annual percentage change of MR was examined using linear regression. To compare MR with Belarus, Ukraine, Czech Republic and Germany, data from the European Mortality Database were used.

**Results:**

MR were the highest in the age 15–19 years (33.7/100,000) and among boys (22.7/100,000). Unintentional injuries including transport accidents, drowning, and suicides (especially in children over 10 years old), were the main cause of death in the analyzed groups. Between 1999 and 2012 annual MR for unintentional injuries declined substantially. MR due to injuries and poisoning in Poland were higher compared with Czech Republic and Germany and lower in comparison with Belarus and Ukraine.

**Conclusions:**

Deaths due to unintentional injuries are still the leading cause of death among Polish children and adolescents. There are differences in death rates between Poland and neighboring countries.

## Introduction

In Poland, similarly to other countries, injuries are one of the leading causes of childhood mortality (WHO and UNICEF 2008). Monitoring trends in mortality is helpful in defining the priorities of public health. Changing patterns of mortality necessitate relevant modifications aimed at the prevention of avoidable deaths (Sidebotham et al. 2014a). Reducing the number of deaths of children and adolescents is a priority of the health policy of many countries (Editorial the Lancet 2014). All the more that the deaths under 19 years of age are mostly avoidable, analysis of trends in deaths due to possibly modifiable factors enables taking preventive measures at the national and local level and contributes to reducing future mortality (Kułaga et al. 2009). Currently, external causes of deaths are the leading cause of avoidable deaths in Europe among children and adolescents (Armour-Marshall et al. 2012; WHO and UNICEF 2008). Although the number of deaths due to external causes among European children has decreased compared with those of the 1990s, the leading causes are transport accidents, drowning and other external causes (Lyons and Brophy 2005; Armour-Marshall et al. 2012). According to the World Health Organization (WHO), every year in the world, as a result of injury and violence about 950,000 children and adolescents below the age of 18 years die, of which nearly 90 % constitute unintentional injuries (WHO and UNICEF 2008). In 2012, in the age group 5–14, injury mortality rate (MR) was 9.0 per 100,000 in the WHO European Region, whereas in other regions varied from 11.3 to 69.2 per 100,000 (WHO 2014a). However, there are still great disparities among European countries in children and adolescents mortality. In the age group 1–19 standardized death rate (SDR) was 5.4 and 22.3 per 100,000 for European Union (EU) members before May 2004 and Commonwealth of Independent States (CIS), respectively (European Mortality Database 2014). The differentiation of mortality is associated with the economic development of the regions. Higher income countries have lower rates of mortality due to external causes than lower and middle-income countries (Patton et al. 2009). According to the World Bank Poland belonged to the middle-income European countries until 2008 and in the 2009 was classified as high-income economies country (World Bank 2016). Poland have moderate mortality rates of children compared with other European countries (Lyons and Brophy 2005; European Mortality Database 2014). Mazur et al. mentioned mortality decline among children and adolescents in Poland from 1990 to 2007 and noticed, in the older age groups, halt of the declining trend in the year 2000 (Mazur and Malinowska-Cieslik 2010). In the period 1999–2006 mortality of Polish children and adolescent due to external causes declined but still accounted for 55 % of all deaths (Kułaga et al. 2009). However, no decline in mortality due to suicide among adolescents aged 10–19 years was observed from 1999 to 2007 (Kułaga et al. 2010). A limitation of the data on trends in children and adolescents mortality after 2007 is the lack of a national perspective (Genowska et al. 2014). In Poland, on national level, exist several measures focusing on preventing children and adolescents mortality due to transport accidents, drowning and poisoning; however, with regard to falls and choking/strangulation existing measures are limited (Malinowska-Cieślik et al. 2012).

The aim of the study was to examine the current pattern and trend of deaths due to external causes among Polish children and adolescents, and to compare trends in child deaths due to injury (including poisoning) in Belarus, Ukraine, Czech Republic and Germany, between 1999 and 2012. We hypothesized that mortality rates due to external causes in Poland decreased, but still are higher compared with European Community countries and lower compared to eastern neighboring countries.

## Methods

### Study population

Anonymised individual records of all deaths of children aged 1–19 years in the years 1999–2012 were obtained from the Central Statistical Office of Poland (http://stat.gov.pl). Each record contained the data: sex, age at death, underlying cause of death coded according to the International Statistical Classification of Diseases and Related Health Problems Tenth Revision (ICD-10). Approval of the Children’s Memorial Health Institute Ethics Committee to conduct the analysis was obtained before study commenced. Data on the size of the population in the years 1999–2001 were obtained from the Local Data Bank (2015) (http://stat.gov.pl/bdl/app/strona.html?p_name=indeks), and in the years 2002–2012 from the Demographics Database (2015) (http://demografia.stat.gov.pl/bazademografia/). Mid-year population estimates were used to calculate MR. In the analyzed datasets causes of death were missing in 48 records, which was due to the doctor’s strike from the years 1997 to 1998, which consisted in not providing the cause of death on the death certificate. These data are omitted in further analysis, assuming that the impact of their absence on the conclusions is small.

### Grouping causes of death

We analyzed data concerning deaths due to external causes coded as V01–Y98 according to the ICD-10, including deaths as a result of unintentional injuries: transport accidents (V01–V99, Y85), accidental drowning and submersion (W65–W74), falls (W00–W19), accidental poisoning (X40–X49), other accidental threats to breathing (W75–W84) and other deaths due to external causes (W20–W64; W85–W99; X00–X39; X58–X59; Y35–Y36; Y40–Y89), due to intentional injuries: intentional self-harm (suicides) (X60–X84, Y87.0) and assault (X85–Y09), as well as deaths due to event of undetermined intent (Y10–Y34, Y87.2, Y89.9).

### Statistical analysis

Statistical analysis was performed with SAS Enterprise Guide 5.1 for Windows software. Mortality rates were calculated per 100,000 inhabitants according to sex and age groups: 1–4, 5–9, 10–14, 15–19 years and for the entire studied group (1–19 years). To compare death rates across age groups and between boys and girls MR 95 % confidence interval (CI) were computed and for testing the equality of MR the overlap of 95 % CIs of the rates was used. The statistical significance of change over time in the mortality rates was investigated by estimating linear regression, in which the response variable was the natural logarithm of mortality, while the explanatory variable—the year. The application of the model in this form enables to determine a fixed annual percent change (APC) in the studied period, according to the equation: ${\text{APC}} = ({\text{exp(}}\beta 1 )- 1) \times 100$, where *β*_1_ is the estimation of the parameter at the variable year (http://seer.cancer.gov/seerstat/WebHelp/seerstat.htm#Trend_Algorithms.htm (National Cancer Institute 2014). The APC 95 % confidence intervals (CI) and statistically significant changes of mortality rates were estimated in the analyzed period. *P* values <0.05 were considered significant. For the purpose of graphic presentation of trends over time, to reduce the annual fluctuations in the mortality rates, 3-year moving averages were used.

Finally, we examined trends in mortality due to injury (including poisoning) in Poland and Poland’s neighboring countries: Belarus, Czech Republic, Germany and Ukraine by plotting rates obtained from the WHO European Mortality Database HFA-MDB (European Mortality Database 2014).

## Results

There were 35,963 children and adolescents aged 1–19 years who died in Poland between 1999 and 2012 (MR—28.8 per 100,000), of whom 24,153 (67.2 %) were boys. In the analyzed period 19,669 children and adolescents died due to external causes, which constituted 54.7 % of all deaths in this age group. External causes deaths rate for boys: 22.7 per 100,000 (95 % CI 22.32–23.06) was significantly higher compared with girls: 8.5 per 100,000 (95 % CI 8.25–8.71) (Table 1). External causes of death constituted: 25.7, 38.8, 47.2 and 69.0 % of all deaths in the age groups: 1–4, 5–9, 10–14, 15–19 years, respectively. Mortality rates due to external causes of death were: 6.8 (95 % CI 6.47–7.17), 6.0 (95 % CI 5.69–6.26), 8.5 (95 % CI 8.16–8.78), and 33.7 per 100,000 (95 % CI 33.12–34.26) for ages 1–4, 5–9, 10–14 and 15–19 years, respectively. Transport accidents were the leading cause and accounted for 44.9 % of deaths due to external causes. Drowning among children aged 1–14 years and suicide among adolescents aged 15–19 years were the second-leading cause of death due to external causes (Table 1).

**Table 1:** Mortality rates per 100,000 population (95% CI) from external causes in Polish children and adolescents aged 1–19 years according to sex, age group and ICD-10 code grouping, 1999–2012

Causes of external deaths (ICD-10)	1–4		5–9		10–14		15–19		1–19	
	Boys	Girls	Boys	Girls	Boys	Girls	Boys	Girls	Boys	Girls
Unintentional injuries	7.3 (6.76–7.76)	4.9 (4.46–5.30)	6.9 (6.48–7.33)	3.9 (3.54–4.19)	8.2 (7.75–8.60)	4.7 (4.34–4.99)	31.8 (31.08–32.62)	11.0 (10.58–11.50)	15.4 (15.05–15.66)	6.6 (6.38–6.79)
Transport accidents (V01–V99, Y85)	2.6 (2.31–2.91)	2.1 (1.83–2.39)	3.9 (3.54–4.17)	2.5 (2.26–2.78)	4.4 (4.06–4.68)	3.0 (2.74–3.26)	21.5 (20.82–22.08)	8.6 (8.23–9.05)	9.5 (9.22–9.69)	4.6 (4.39–4.73)
Accidental drowning and submersion (W65–W74)	1.8 (1.56–2.06)	1.0 (0.77–1.14)	1.6 (1.35–1.75)	0.4 (0.27–0.48)	2.0 (1.75–2.16)	0.7 (0.60–0.86)	4.3 (4.05–4.62)	0.6 (0.51–0.73)	2.6 (2.48–2.73)	0.6 (0.59–0.71)
Falls (W00–W19)	0.6 (0.45–0.73)	0.3 (0.18–0.38)	0.2 (0.12–0.26)	0.1 (0.06–0.18)	0.4 (0.29–0.48)	0.1 (0.05–0.15)	1.6 (1.39–1.73)	0.4 (0.31–0.48)	0.8 (0.69–0.82)	0.2 (0.19–0.27)
Accidental poisoning (X40–X49)	0.4 (0.25–0.47)	0.3 (0.16–0.36)	0.2 (0.12–0.26)	0.2 (0.09–0.22)	0.2 (0.17–0.31)	0.3 (0.22–0.39)	0.9 (0.81–1.07)	0.7 (0.56–0.79)	0.5 (0.42–0.53)	0.4 (0.33–0.43)
Other accidental threats to breathing (W75–W84)	0.6 (0.45–0.73)	0.4 (0.31–0.57)	0.3 (0.19–0.35)	0.2 (0.12–0.26)	0.3 (0.25–0.42)	0.1 (0.07–0.18)	0.6 (0.49–0.70)	0.2 (0.11–0.22)	0.4 (0.40–0.50)	0.2 (0.17–0.24)
Other unintentional injuries (W20–64; W85–99; X00–39; X58–59; Y35–36; Y40–89)	1.3 (1.09–1.52)	0.8 (0.66–1.02)	0.8 (0.70–0.99)	0.5 (0.39–0.62)	0.9 (0.75–1.03)	0.4 (0.31–0.50)	3.0 (2.73–3.20)	0.5 (0.43–0.64)	1.6 (1.52–1.72)	0.5 (0.49–0.60)
Intentional injuries	0.2 (0.15–0.34)	0.2 (0.12–0.30)	0.2 (0.10–0.24)	0.2 (0.12–0.26)	2.1 (1.87–2.30)	0.8 (0.66–0.93)	15.9 (15.33–16.41)	3.1 (2.88–3.37)	5.8 (5.58–5.96)	1.3 (1.22–1.40)
Intentional self–harm (suicide) (X60–X84, Y87.0)	n/a	n/a	0.0 (0.00–0.04)	0.0 (–0.01–0.03)	1.9 (1.71–2.12)	0.6 (0.52–0.76)	15.1 (14.55–15.61)	2.7 (2.47–2.93)	5.4 (5.21–5.57)	1.1 (0.97–1.13)
Assault (X85–Y09)	0.2 (0.15–0.34)	0.2 (0.12–0.30)	0.1 (0.09–0.21)	0.2 (0.11–0.25)	0.2 (0.11–0.23)	0.2 (0.10–0.22)	0.8 (0.67–0.91)	0.4 (0.34–0.52)	0.4 (0.33–0.43)	0.3 (0.22–0.30)
Event of undetermined intent (Y10–Y34, Y87.2, Y89.9)	0.6 (0.43–0.71)	0.4 (0.28–0.52)	0.5 (0.36–0.58)	0.3 (0.18–0.35)	0.7 (0.61–0.86)	0.4 (0.26–0.45)	3.6 (3.33–3.84)	1.1 (0.97–1.27)	1.6 (1.47–1.66)	0.6 (0.53–0.65)
All causes of external deaths	8.1 (7.54–8.60)	5.5 (5.04–5.94)	7.5 (7.10–7.99)	4.3 (3.98–4.67)	11.0 (10.51–11.49)	5.8 (5.45–6.18)	51.3 (50.33–52.28)	15.3 (14.74–15.83)	22.7 (22.32–23.06)	8.5 (8.25–8.71)

*95% CI* 95 % confidence interval, *n/a* not applicable

Deaths due to transport accidents were mainly related to injury to car users (V40–V49; 43.2 %) and injury to pedestrians (V01–V09; 27.7 %). In the case of drowning, deaths followed as a result of submersion and drowning in natural waters (W69–W70; 64.5 %), other determined (W73) and undetermined (W74) submersion and drowning (32.0 %), and as a result of drowning in the bathtub or swimming pool (W65–W68; 3.5 %). In contrast, deaths due to intentional self-harm (suicide), in the majority of cases (88.9 %), were result of hanging (X70). Mortality rate due to intentional causes of death (mostly assault and few cases of suicide) was 0.2 per 100,000 (95 % CI 0.16–0.24) in age 1–9 years. In children aged 10–14 years intentional causes mortality rate was: 0.8 (95 % CI 0.66–0.93) and 2.1 (95 % CI 1.87–2.30) in girls and boys, respectively, whereas in adolescents (15–19 years of age) the rates were the highest: 3.1 (95 % CI 2.88–3.37) and 15.9 (95 % CI 15.33–16.41) in girls and boys, respectively (Table 1). Suicide constituted 89 and 94 % of all intentional causes of death in ages: 10–14 and 15–19 years, respectively. Approximately 7 % of all deaths due to external causes were classified as event of undetermined intent. Mortality rates due to event of undetermined intent were: 0.5 (95 % CI 0.43–0.52), 1.1. (95 % CI 0.97–1.27), 3.6 (95 % CI 3.33–3.84) in the case of boys and girls aged 1–14 years, girls aged 15–19 years and boys aged 15–19 years, respectively (Table 1).

**Fig. 1:**
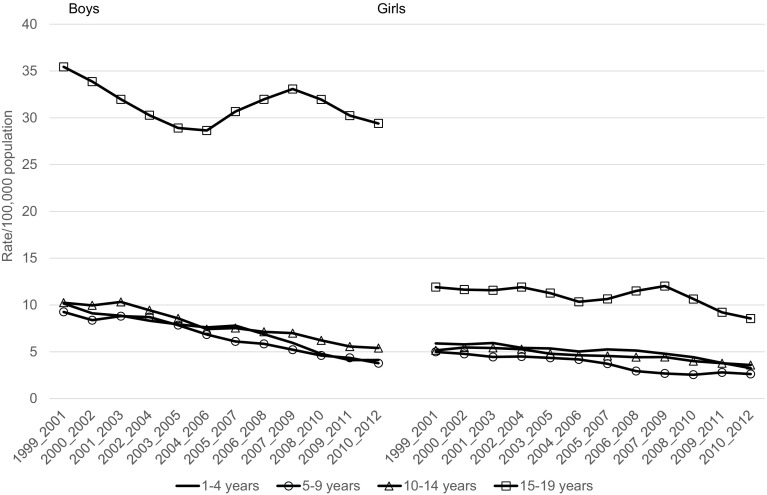
Trends in 3-year moving averages of mortality rates due to unintentional injuries among boys and girls aged 1–4, 5–9, 10–14 and 15–19 years, 1999–2012, Poland

**Fig. 2:**
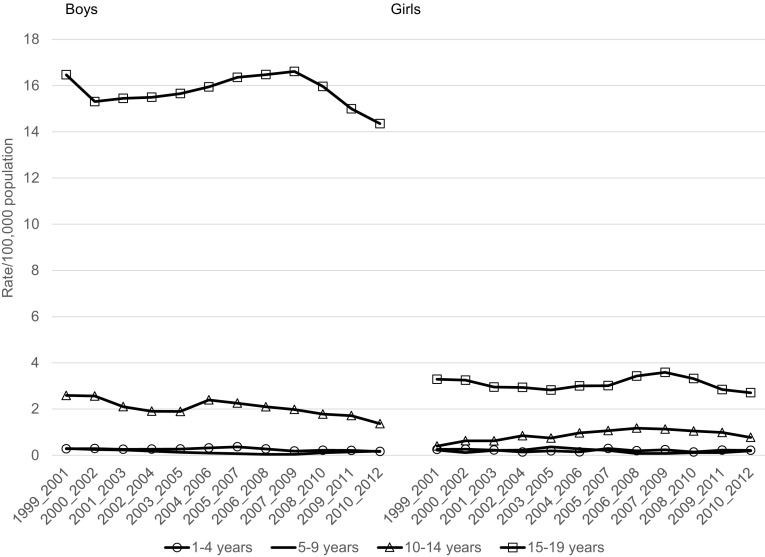
Trends in 3-year moving averages of mortality rates due to intentional injuries among boys and girls aged 1–4, 5–9, 10–14 and 15–19 years, 1999–2012, Poland

**Fig. 3:**
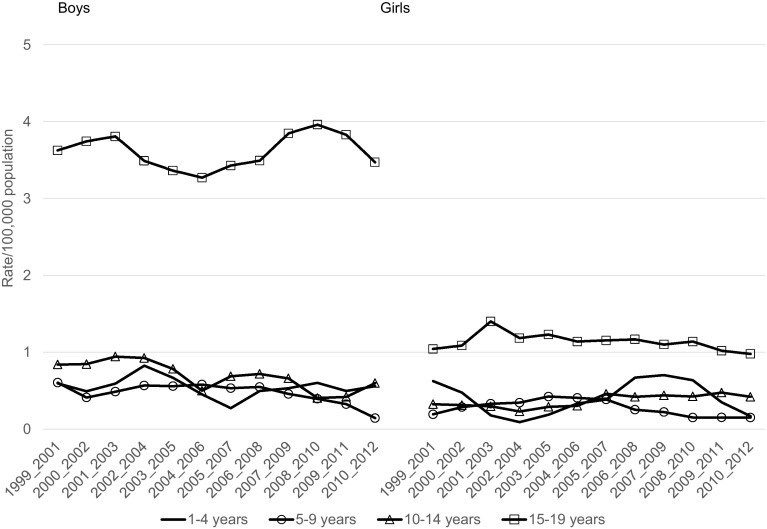
Trends in 3-year moving averages of mortality rates due to deaths related to events of undetermined intent among boys and girls aged 1–4, 5–9, 10–14 and 15–19, 1999–2012, Poland

**Fig. 4:**
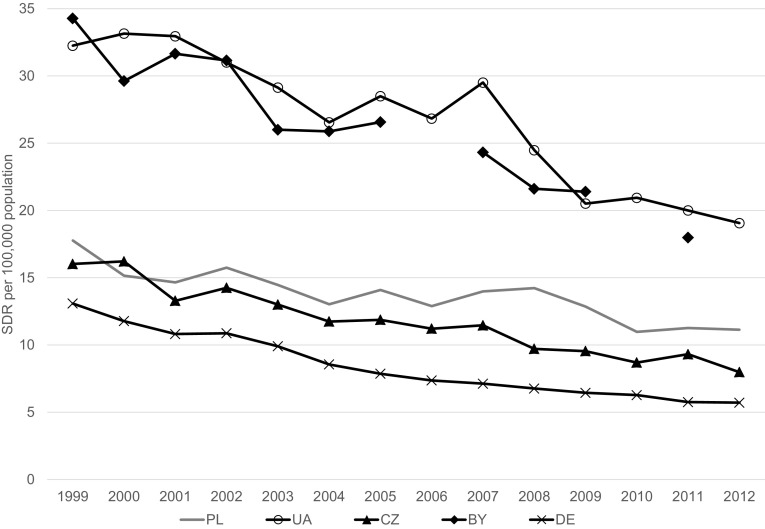
Age-standardized death rates from external causes of injury and poisoning of children and adolescent aged 1–19 years in Belarus (BY), Czech Republic (CZ), Germany (DE), Poland (PL), Ukraine (UA), 1999–2012

In the age group 1–19 years, the mortality rate due to external causes declined between 1999 and 2012 from 28.8 to 17.7 per 100,000 and from 9.8 to 6.1 per 100,000, respectively, for boys and girls (Table 2). Mortality rates due to unintentional injury significantly and substantially declined over the period under the study with the exception of boys aged 15–19 years, for whom the decline was nonsignificant (Table 2; Fig. 1). In the case of deaths due to intentional injuries (suicide, assault) statistically significant decrease in mortality was observed in boys aged 10–14 years and 1–19 years. In the other groups observed changes in the mortality rates were statistically nonsignificant (Table 2; Fig. 2). The mortality rates due to event of undetermined intent in the analyzed age groups, both for boys and girls, generally did not change over the period 1999–2012 (Table 2; Fig. 3). The mortality due to injury and poisoning among children and adolescents declined in all the compared countries (Fig. 4). Death rate in Poland was higher compared to Czech Republic and Germany and lower compared to Ukraine and Belarus (Fig. 4).

**Table 2:** Change of mortality rates per 100,000 population due to external causes of death according to sex and age group in Poland from 1999 to 2012

Age group	Sex	Initial-1999	Final-2012	APC	95 % CI	*p*
Accidental injury (unintentional)						
1–4	Boys	13.0	4.4	−7.76	−9.85 to −5.63	<0.001
	Girls	6.3	2.1	−5.44	−8.04 to −2.76	0.001
5–9	Boys	11.1	3.0	−8.21	−9.81 to −6.57	<0.001
	Girls	5.7	2.2	−6.48	−8.42 to −4.49	<0.001
10–14	Boys	11.3	5.7	−5.76	−7.13 to −4.36	<0.001
	Girls	5.2	3.5	−3.34	−4.64 to −2.02	<0.001
15–19	Boys	39.4	28.7	−1.27	−2.61 to 0.08	0.063
	Girls	13.3	8.5	−2.56	−4.38 to −0.70	0.011
1–19	Boys	20.3	11.5	−3.37	−4.38 to −2.35	<0.001
	Girls	8.1	4.3	−3.72	−5.16 to −2.26	<0.001
Assault and suicide (intentional)						
1–4	Boys	0.3	0.0	−4.25	−12.01 to 4.20	0.282
	Girls	0.1	0.2	0.88	−5.67 to 7.89	0.776
5–9	Boys	0.4	0.2	−4.91	−11.10 to 1.71	0.127
	Girls	0.3	0.3	−0.49	−10.49 to 10.63	0.919
10–14	Boys	2.4	1.3	−4.12	−7.75 to −0.34	0.035
	Girls	0.3	0.6	6.88	−0.18 to 14.43	0.055
15–19	Boys	19.3	14.8	−0.82	−1.92 to 0.30	0.135
	Girls	3.4	3.2	−0.66	−3.23 to 1.98	0.594
1–19	Boys	6.9	4.7	−1.7	−2.89 to −0.50	0.010
	Girls	1.2	1.3	−0.21	−2.37 to 1.99	0.835
Events of undetermined intent						
1–4	Boys	0.9	0.6	−1.31	−9.33 to 7.41	0.740
	Girls	0.5	0.1	−5.54	−15.86 to 6.05	0.302
5–9	Boys	0.9	0.0	−7.51	−14.61 to 0.18	0.055
	Girls	0.1	0.2	1.25	−5.91 to 8.96	0.716
10–14	Boys	0.8	0.9	−4.27	−9.77 to 1.58	0.135
	Girls	0.3	0.3	3.92	−3.07 to 11.42	0.252
15–19	Boys	3.3	3.7	0.14	−1.58 to 1.89	0.863
	Girls	1.0	1.1	−0.05	−4.12 to 4.19	0.980
1–19	Boys	1.6	1.4	−1.42	−3.13 to 0.31	0.099
	Girls	0.5	0.5	−0.49	−4.05 to 3.21	0.774
All causes of external deaths						
1–4	Boys	14.2	5.0	−7.26	−9.13 to −5.35	<0.001
	Girls	6.9	2.4	−5.06	−7.66 to −2.39	0.002
5–9	Boys	12.4	3.2	−8.23	−9.92 to −6.50	<0.001
	Girls	6.1	2.8	−6.14	−8.23 to −4.00	<0.001
10–14	Boys	14.6	8.0	−5.32	−6.45 to −4.17	<0.001
	Girls	5.8	4.4	−1.85	−3.24 to −0.45	0.014
15–19	Boys	62.1	47.2	−1.04	−2.12 to 0.05	0.059
	Girls	17.8	12.8	−2.00	−3.73 to −0.24	0.029
1–19	Boys	28.8	17.7	−2.82	−3.74 to −1.89	<0.001
	Girls	9.8	6.1	−2.96	−4.38 to −1.51	0.001

*APC* annual percent change, *95 % CI* 95 % confidence interval

## Discussion

The presented analysis shows the characteristic and trends of deaths due to external causes among Polish children and adolescents. Our nationwide data showed that mortality due to external causes in general and those unintentional, decreased substantially and significantly in each analyzed age group, except boys aged 15–19 years. Results of the analysis are consistent with the trend of mortality reported in other countries (Gijzen et al. 2014; Pan et al. 2006). It has been shown that the share of deaths due to external causes increased with age among both boys and girls. In the present study we observed that 54.7 % of all deaths were due to external causes; this percentage is similar to England and Wales where external causes accounted for more than half of all deaths of adolescents (Sidebotham et al. 2014b) and also male deaths in the age 10–24 years worldwide (Patton et al. 2009). The mortality rate due to injury was higher in Polish boys compared with girls, reaching peak level in the age 15–19 years, which is in line with other reports (Fraser et al. 2014; Gissler et al. 2009). The difference in mortality between boys and girls was similar to the Scandinavian countries (Gissler et al. 2009), thus the lowest for younger children (1–9 years) and increasing with age. In the case of Polish boys aged 15–19 years, mortality was three times higher compared with girls, similarly to data from England and Wales (Sidebotham et al. 2014b).

In Poland, like in most countries, transport accidents are the leading cause of death among children and adolescents aged 10–19 years (Patton et al. 2009). The reasons for decreasing trend of mortality due to transport accidents are multifactorial. In Poland it can be attributed to legislation, i.e., the obligation of fastening seat belts in the car (in Poland since 1991 the obligation of fastening seat belts was extended also to the back seats and all roads—not only front seats and roads outside built-up areas as in 1983), the obligation of using car seats for transporting children under the age of 12 years who are shorter than 150 cm, limiting speed, the recommendation of children wearing a helmet when riding a bicycle. In addition, actions aimed at improving the environment, i.e., improving the network of roads and road signs, introducing new safety standards for vehicles, education on road traffic, developing care that deals with emergency cases (emergency medicine) can have a positive impact on reducing incidences of transport accidents and improving health outcomes of accident victims (Pan et al. 2006). In recent years, in most European countries the number of unintentional transport accidents has decreased (Armour-Marshall et al. 2012; De Grande et al. 2013). However, in Poland, transport accidents, especially accidents of car users and involving pedestrians, are still the leading cause of deaths in the age 10–19 years. Sweden, where the promotion of safety in society started in the 1950s, has the lowest mortality rate due to transport accidents (UNICEF 2001). This is in connection with implementing urban planning and legislative solutions, which are conducive to safety through, e.g., the creation of areas without car traffic or separating traffic of cars, bicycles and pedestrians, as well as the creation of safe places where children can spend their free time (WHO and UNICEF 2008). Using the best practices of countries that have the lowest rate of deaths due to accidents seems to be the right approach in taking appropriate preventive measures.

In Poland, drowning is the second cause of death due to unintentional causes in the age 1–14 years. A similar situation exists in Europe (Armour-Marshall et al. 2012), Canada (Pan et al. 2006) and the United States (Centers for Disease Control and Prevention 2015). In Poland, the highest rate of death due to drowning was among boys aged 15–19 years; whereas, in the case of girls, the age group 1–4 years was the most vulnerable. In many countries (WHO 2014b) drowning of children aged 1–4 years is more common compared with older children. The reduction of the mortality rate due to drowning can be associated with the introduction of effective interventions as: increased parental supervision of children when they are in the water or by the water, greater control of water lifeguards at water reservoirs, using instruments that facilitate safe movement in water, safeguarding water reservoirs, educating the public regarding the dangers to children near water reservoirs, and first aid education (Wilson et al. 2006). In addition, children’s swimming lessons appears to be an effective strategy for preventing drowning of children (Brenner et al. 2009).

In Poland, in the category of deaths due to intentional injuries, suicide is the primary cause. Suicide was the third cause of death in the age 10–14 years and the second cause among 15–19 years old, which is in line with global reports (Hawton et al. 2012). In Poland, similarly to England and Wales, suicide death rates are the highest among older adolescents and boys (Windfuhr et al. 2013; Napieralska et al. 2010). Between the 1980s and 1990s an increase in the rate of suicides among adolescents in many European countries was observed (Rutz and Wasserman 2004). Suicides of children and adolescents raise a lot of controversies about the causes. This phenomenon is quite complex. The risk factors associated with youth suicides include: mental disorders, abuse of psychoactive substances, especially alcohol, disruption of relationships associated with family breakdown, social exclusion associated with unemployment, the role of the male in society (McClure 2001; Brent et al. 1994).

In our dataset, a fixed, almost 7 % share of deaths due to undetermined intent was found, which is higher compared with EU countries average (French Institute for Public Health Surveillance 2008). Event of undetermined intent may suggest, like in Finnish studies (Ohberg and Lonnqvist 1998), the masking of suicide. Hence, monitoring the mortality due to suicide should also take into account the observations of trends of deaths due to events of undetermined intent (Kułaga et al. 2010). In Estonia more than 10 % of deaths in children (0–14 years) due to undetermined intent indicate an underestimation of deaths due to intentional injury, and because mostly these deaths involved children younger than 1 year, the authors concluded that it may be as a result of homicide (Väli et al. 2007). Therefore, it is important to take actions aimed at obtaining accurate information regarding the cause of death, paying particular attention to the intent of event leading to fatality.

From public health perspective, the results of our analysis show that Poland belongs to countries, which have medium level of children and adolescents mortality due to external causes. Presented death trends in neighboring Germany and Czech Republic may lead to the conclusion that there is room for improvement. With regard to the unintentional injuries, measures aimed at improvement of affordability of available safety devices, which is still lower in Poland compared with many European Child Safety Alliance countries (European Child Safety Alliance 2012), are recommended. Also establishment of childhood injury register in Poland would enable to identify the underlying mechanisms of accidents and target injury prevention in a qualified manner. With regard to self-inflicted injuries a comprehensive nationwide program of suicide prevention, addressed to the youth should be implemented in Poland.

### Limitation of the study

The limitation of the study is relatively high mortality rate due to event of undetermined event among Polish adolescent (especially boys aged 15–19 years) which may mask other external causes of death especially suicide.

### Strong point

Analysis included all death records between 1999 and 2012 coded uniformly according to ICD-10.

Mortality data and trends were analyzed using strict statistical methodology.

Comparison included relevant to Poland neighboring countries.
